# Canonical correlation analysis (CCA) of anthropometric parameters and physical activities with blood lipids

**DOI:** 10.1186/s12944-017-0630-3

**Published:** 2017-12-08

**Authors:** Na Yu, Qingjun Zhang, Lan Zhang, Tianjing He, Qing Liu, Sheng Zhang

**Affiliations:** 1 Department of Epidemiology and Medical Statistics, Wuhan University, Hubei, China; 2 The Center for Disease Control and Prevention of Hubei Province, 2 Zhuodaoquan North Road, Wuhan, Hubei 430000 China; 3 Department of Epidemiology and Medical Statistics, Nantong University, Jiangsu, China

**Keywords:** Anthropometric parameters, Physical activities, Blood lipids, Canonical correlation analysis

## Abstract

**Background:**

Anthropometric parameters and physical activities are significant factors influencing lipid levels, but few research have demonstrated the effect of amount of activities on lipid levels. Our research analyzed and explored this relationships.

**Methods:**

A multi-stage stratified sampling method was used to select the investigation subjects in Hubei, China. A questionnaire survey, physical measurements and biochemistry tests (including total cholesterol, high low-density lipoprotein cholesterol, high-density lipoprotein cholesterol and triacylglycerol) were conducted using *CCA* analysis.

**Results:**

The first canonical correlation of the four biochemistry tests and anthropometric parameters with physical activities was 0.44 (*P* < 0.0001). Grouping by sex and areas, the first canonical correlation were 0.51 (*p* < 0.0001), 0.43 (*p* < 0.0001), 0.39 (*p* < 0.0001) and 0.45 (*p* < 0.0001). By CCA, blood lipids were negatively correlated with occupation activity, and positively associated with waistline, body mass index (BMI), sleep time, static behavior, and age.

**Conclusions:**

CCA could be an efficient method to find out the most influential factors on exposure and outcome variables. Blood lipid had significant but moderate association with physical activities and anthropometric parameters. Waistline, BMI and occupation activity function as major influences on lipids.

**Trial registration:**

Identifying number: 2,013,001. Date of trial registry: 8st Oct 2012.

**Electronic supplementary material:**

The online version of this article (10.1186/s12944-017-0630-3) contains supplementary material, which is available to authorized users.

## Background

Accumulated research had shown that lipoprotein (LP(a)) was an important risk factor of ischemic stroke and cardiovascular diseases [1–4], high LP(a) as an independent but modest risk factor in ischemic stroke. This becomes especially evident in younger stroke populations. However, based on published research, we knew that there was a relationship between physical activities and blood lipid [5–7], the intensity of physical activity may be a more important determinant of LDL-C in children than the energy spent on physical activity. At the same time, individual factors couldn’t be ignored to have an effect on lipid, and study provides some evidence to sharpen the target levels for glycemia and BMI among patients with low HDL-C and high TG. For these patients, the target glycemia should be around 90 mg/dl and BMI 25 kg/m2 [8]. Physical activities with individual anthropometric parameters affecting or not affecting blood lipids, isolated through canonical correlation analysis, were consistent with evidence of these kinds of associations in the literatures [7, 9]. The purpose of the research is to better understand the role and the influential degree that anthropometric parameters and physical activity have played on lipids.

*CCA* is a multivariate statistical model that facilitates the study of linear interrelationships between two sets of variables: one set of variables is referred to as independent and the other as dependent; a composite score is formed for each set. *CCA* develops a canonical function that maximizes the correlation between the two composite variables [10]. Additionally, *CCA* develops as many functions as there are variables in the smaller variable set; each function is independent from the others so that they represent different relationships among the sets of dependent and independent variables [11].

## Methods

### Study design and participants

In the 2013 non-communicable disease (*NCD*) surveillance of Hubei province in China, we extracted 6000 families from 120 villages of 10 Surveillance Points. The final number of valid samples was 5878. Subjects being investigated were the inhabitants age 18 and over in all surveillance spots, 2362 males and 3516 females, 1753 in urban and 4125 in rural. Centralized and family investigation were carried out while the survey information was collected by investigators through on-site and face to face inquiry method. The content of surveillance included questionnaire (sex, age, address, occupation activity (high and moderate intensive labor at least for 10 min), transportation activity (walk or bike at least for 10 min), leisure time activity (high and moderate intensive exercise at least for 10 min), static behavior (television, computer, phone or reading) and sleep time), physical measurement (height, weight and waistline) and biochemistry test (total cholesterol *(TC),* high low-density lipoprotein cholesterol *(HLDL-C),* high-density lipoprotein cholesterol *(HDL-C)* and triacylglycerol *(TG)*).

### Data analysis

To support our *CCA* findings we described our data by mean, standard deviation, median and quartile to show the distributions and numerical characteristics. We used *CCA* to make a full analysis, physical activitiy time and anthropometric parameters as independent variables while blood lipids as dependent variables, and we also recorded the loading and cross loadings of the two variable sets. To make a deep research, we stratified our samples by sex and area for four strata (Female, Male, urban and rural) and investigated the relationship with lipids.

Pearson’s correlation and multiple linear regressions were used to examine the relationship between lipids and physical activities with anthropometric parameters and to find the function of individual variables have played on one’s lipids.

When correlation significance level was less than 5% and redundancy coefficient greater than 0.10, we recognized the responding data as meaningful result. All analyses were performed using Statistical Analysis Software (SAS), version 8.2(SAS institute, Cary, NC).

## Results

Additional file 1: Table S1 presents the results of descriptive statistics for anthropometric parameters, physical activities and blood lipids, and the mean age of investigated persons was 50.96. Compared to normal range, all variables except age were included.

Pearson’s correlation coefficient and regression coefficient between age, BMI, waistline, kinds of physical activity time and four biochemistry indexes were shown in Table 1. All variables except transportation activity time were correlated with *HDL-C* and *TG*. All variables except leisure activity time were correlated with *LDL-C* and *TC*. While after regression analysis, only age and waistline had a positive effect on *LDL-C* and *TC*. *BMI*, waistline, occupation activity and sleep time show their function on TG in regression analysis.

**Table 1:** Pearson’s correlation coefficient, r (*p*-value), and regression analysis, **β**(*p*-value) between the anthropometric parameters, physical activities and blood lipids from Hubei province in 2013, *n* = 5878

	HDL-C R	β	LDL-C R	β	TG R	β	TC R	β
Intercept	–	2.196*	–	1.120*	–	−1.042*	–	2.77*
Age	0.083*	0.004*	0.218*	0.011*	0.101*	−0.001	0.242*	0.013*
BMI, Kg/m^2^	−0.318*	−0.023*	0.184*	0.008	0.386*	0.036*	0.154*	0.001
Waistline, cm	−0.291*	−0.006*	0.234*	0.013*	0.421*	0.023*	0.222*	0.016*
Occupation activity, hours/week	0.153*	0.003*	0.036**	0.002	−0.104*	−0.006*	0.044*	0.002
Transportation activity, hours/week	0.015	−0.000	0.047*	0.002	0.008	0.000	0.041**	0.002
Leisure time activity, hours/week	−0.093*	−0.004**	0.012	0.004	0.063*	0.002	0.001	0.001
Static behavior, hours/day	−0.152*	−0.016*	−0.048*	−0.002	0.063*	0.009	−0.072*	−0.008
Sleep time, hours/day	0.058*	0.009**	−0.065*	−0.008	−0.105*	−0.027**	−0.066*	−0.012

*BMI* body mass index, *TC* total cholesterol, *HLDL-C* high low-density lipoprotein cholesterol, *HDL-C* high-density lipoprotein cholesterol, *TG* triacylglycerol

*P < 0.001, **P < 0.01

Table 2 shows direct result for the correlation between the two variable sets, which presented the canonical correlation coefficients and the redundancy indices for the research. There were four function correlations because the dependent set contained the minimum number of four variables, and the correlations for each successive function were 0.44, 0.26, 0.09 and 0.04. According to the screening criterions, the last one were not statistically significant (*P* < 0.05, F-test) and the first redundancy index for all functions was much larger than another 2 indexes. In this way, the first function correlation coefficient could be utilized for analysis in the context of this study.

**Table 2:** Canonical correlation analysis of anthropometric parameters, physical activities and blood lipid from Hubei province in 2013, *n* = 5878

Canonical variates	Canonical Correlation	F-statistic	*P*-value	Redundancy Index, R _Y/X_
Variate-1	0.44	55.63	<0.0001	0.1897
Variate-2	0.26	23.04	<0.0001	0.0684
Variate-3	0.09	5.14	<0.0001	0.0086
Variate-4	0.04	2.19	0.0527	0.0019

Considering different physical activities’ time and anthropometric parameters in male and female, urban and rural, we calculated the first canonical correlation coefficient between blood lipid and anthropometric parameters with physical activities in Table 3, and they were 0.51, 0.43, 0.39 and 0.45.

**Table 3:** The first canonical correlation analysis of anthropometric parameters, physical activities and blood lipid grouping by sex and area from Hubei province in 2013, *n* = 5878

Groups	The first canonical Correlation	F-statistic	*P*-value
Male (*n* = 2362)	0.51	27.25	<0.0001
Female (*n* = 3516)	0.43	34.38	<0.0001
Urban (*n* = 1753)	0.39	12.79	<0.0001
Rural (*n* = 4125)	0.45	43.89	<0.0001

To find out key factors in each group of variables, we presented the loadings and cross loadings for the first canonical function in Table 4. In the loadings of the variables for function 1, the most effective factor was waistline (loading: 0.920) followed by *BMI* (loading: 0.878), occupation activity (loading: −0.242), sleep time (loading: 0.178), static behavior (loading: 0.175) and age (loadings: 0.133). For lipids, the results of the loadings indicated that *HDL-C* and *TG* similarly contributed to the first canonical function, so as to *LDL-C* and *TC*, though whose function was relatively less. So, blood lipids were negatively correlated with occupation activity, and positively associated with waistline, BMI, sleep time, static behavior, and age, in above order.

**Table 4:** The loadings and cross loadings of the variables for the first canonical function in canonical correlation analysis, *n* = 5878

Variables	Loadings	Cross loadings
Independent variables		
Age	0.133	0.058
BMI, Kg/m^2^	0.878	0.383
Waistline, cm	0.920	0.401
Occupation activity, hours/week	−0.242	−0.105
Transportation activity, hours/week	0.031	0.014
Leisure time activity, hours/week	0.117	0.051
Static behavior, hours/day	0.175	0.076
Sleep time, hours/day	0.178	−0.077
Dependent variables		
HDL-C	−0.771	−0.336
LDL-C	0.427	0.186
TG	0.722	0.314
TC	0.403	0.176

*BMI* body mass index, *TC* total cholesterol, *LDL-C* low-density lipoprotein cholesterol, *HDL-C* high-density lipoprotein cholesterol, *TG* triacylglycerol

## Discussion

As CCA uses information from all the variables in the exposure and outcome variable sets and maximizes the estimation of the relationship between the two sets, [10] *CCA* may assess the effects of the physical activities and anthropometric parameters on lipids in a more efficient way. Because of limiting the inefficiencies that may accompany conventional multiple testing, *CCA* could help to reduce type-1 error (an error for refusing the truth, usually represented by “α”) and add accuracy to its results. Furthermore, in *CCA* the latent variable approach, as used, helped to avoid multicollinearity (the presence of precise or highly correlated relationship between the variables in linear regression model make the model estimation distort or difficult to estimate accurately.) [12].

For its particularity, *CCA* carried out based on multiple independent variables and multiple dependent variables, so we choose the method to study the correlation between lipids and physical activities with anthropometric parameters. After calculations, we found that lipids had significant but moderate associations with physical activities and anthropometric parameters. The finding of this reminds us that we should not ignore the role of physical activities and anthropometric parameters have played on our physical condition. In addition to providing an assessment of the association between two sets of variables, the application of *CCA* could narrow down, in some extent, exposure (physical activities and anthropometric parameters) and outcome variables (lipids) that might contribute to the relationship based on the variable loadings. For example, we may explore the function of waistline and *BMI* on *HDL-C* or *TG* according to our loading results. Thus, *CCA* could be a method to get the most influential factors in both exposure and outcome variables, which may provide more accurate information about the correlation between one’s exposure and outcome and would be a basis for another deep research.

In Table 4, we found that the four lipids indexes were highly correlated. Indicators containing the four indexes capture more information, which could be more effective to predict future health outcomes than a single one. For example, *HDL-C*, accompany with *LDL-C*, *TG* and *TC*, can provide abundant information for the influence of health outcomes had devoted to the future body circumstance, health and development. The same conclusion is drawn that waistline and *BMI*, as significant impact factors of *HDL-C*, indicates keeping a normal waistline or a suitable fit can reduce the risk of angiocardiopathy [1, 13]. High *BMI* was more strongly related to adverse cardiovascular biomarker levels than physical inactivity. However, within *BMI* categories, physical activity was generally associated with more favorable cardiovascular biomarker levels than inactivity [14]. In previously sedentary healthy adults, a lifestyle physical activity intervention is as effective as a structured exercise program in improving physical activity, cardiorespiratory fitness, and blood pressure [15]. In this way, we may conclude that the indicators of our blood lipids may act as a direction for our future lifestyle and a warning to disease that may happen.

Pearson’s correlation coefficients showed that age, occupation activity, and sleep time were significantly positively associated with *HDL-C*, whereas, expectedly, *BMI*, waistline, leisure time activity and static behavior was strongly negatively associated with it. The individual multiple linear regression analyses also depicted virtually identical results, excepted for transportation activity, all other predictors had significant b-coefficients (*P* < 0.05). While the relevant result had shown in Komal’s paper that leisure time physical activity leads to improvement of lipid profile and reduction of obesity as a major atherosclerosis risk factor. It is therefore recommended to implement community-based interventions for promoting leisure time physical activity [16]. While the loading of leisure time activity was 0.117, which played a weak but positive function on lipids. The reason of this phenomenon may be explained that the role of leisure time physical activity had been changed by other variables in Pearson’s correlation analysis.

Grouping by sex and area, we can know from the result that lipids tend to have a strong correlation with physical activity and anthropometric parameters no matter in male and female population or people living in rural and urban. Activity level had a beneficial association with lipid profiles in both sexes, while dietary fat intake was positively associated with *LDL-C* in males and with *HDL-C* in females. In sum, diet, adiposity, and physical activity predict variability in lipid profiles in this adolescent Filipino population [17]. During research, we found that a stronger correlation between lipids and physical activity with anthropometric parameters had been shown in male, and the same conclusion happened in rural population. In combination with the existed results from this paper, we know that occupation activity has played a vital role on the influence of lipids, and then we may explain above situations by traditional accustoms in China. In our country, men tend to act as the main laborers in a family and rural inhabitants tend to perform more labor-intensive work. Besides, the average age of subjects we surveyed was 50.96, while this can be a confounding factor to the research. Male at this age, because of body conditions, were still at work and female were ready to retire no matter in urban or rural places. Compared to people who live in countryside, urban population, tend to perform little to none physically demanding work. Although in different population, the first canonical correlation coefficients were almost same. Considering the national conditions in China, it’s not difficult to get that the difference of sex and area couldn’t change the relationship between lipids and physical activities with anthropometric parameters.

## Conclusion

*CCA* can work as an efficient method to find out the most influential factors in both sets of variables and assess the association between blood lipids and physical activities with anthropometric parameters. The function in deducing the influential variables provides a platform for further research, a basis for future body movements and physical conditions and a direction for healthy development. BMI and waistline played evident roles in *HDL-C* and *TG*. In physical activity, occupation activity time contributed most to lipids.

## Additional file

Additional file 1: Table S1. Descriptive statistics for anthropometric parameters, physical activities and blood lipids from Hubei province in 2013, *n* = 5878. (DOCX 15 kb)

## Abbreviations

BMI: Body mass index
CCA: Canonical correlation analysis
HDL-C: High-density lipoprotein cholesterol
LDL-C: Low-density lipoprotein cholesterol
SD: Standard deviation
TC: Total cholesterol
TG: Triacylglycerol
