# Supplementary material for: Canonical correlation analysis (CCA) of anthropometric parameters and physical activities with blood lipids
Source: Lipids Health Dis. 2017 Dec 8;16:236. doi: 10.1186/s12944-017-0630-3 (PMC5721384; doi:10.1186/s12944-017-0630-3)
Supplement: Additional file 1: Table S1. — Descriptive statistics for anthropometric parameters, physical activities and blood lipids from Hubei province in 2013, n = 5878. (DOCX 15 kb) [file 12944_2017_630_MOESM1_ESM.docx]

**Table S1** Descriptive statistics for anthropometric parameters, physical activities and blood lipid from Hubei province in 2013, n = 5878.

| variables | Mean(SD) | Median(Quartile) |
| --- | --- | --- |
| anthropometric parameters |  |  |
| age | 50.96(14.00) | 51.27(19.32) |
| BMI, Kg/m^2^ | 23.93(3.48) | 23.63(4.67) |
| Waistline, cm | 83.17(10.22) | 83.00(14.00) |
| physical activities |  |  |
| occupation activity, hours/week | 12.92(14.59) | 7.00(16.67) |
| transportation activity, hours/week | 3.65(5.18) | 2.00(5.00) |
| leisure time activity, hours/week | 1.12(3.35) | 0.00(0.00) |
| static behavior, hours/day | 4.85(2.60) | 4.00(3.00) |
| sleep time, hours/day | 7.54(1.54) | 8.00(1.00) |
| blood lipids |  |  |
| HDL-C, mmol/l | 1.39(0.41) | 1.34(0.53) |
| LDL-C, mmol/l | 2.89(0.87) | 2.82(1.13) |
| TG, mmol/l | 1.43(1.13) | 1.13(0.88) |
| TC, mmol/l | 4.70(0.98) | 4.64(1.29) |

TC: total cholesterol; LDL-C: low-density lipoprotein cholesterol;

HDL-C: high-density lipoprotein cholesterol；TG: triacylglycerol.

BMI: body mass index; SD: standard deviation.
